# The role of Community Mobilization in maternal care provision for women in sub-Saharan Africa- A systematic review of studies using an experimental design

**DOI:** 10.1186/s12884-017-1458-6

**Published:** 2017-08-29

**Authors:** Choolwe Muzyamba, Wim Groot, Sonila M. Tomini, Milena Pavlova

**Affiliations:** 1 UNU MERIT, Boschstraat, 246211 AX Maastricht, The Netherlands; 2 A9 Marshlands Village Box 32379, Lusaka, Zambia; 3 Department of Health Services Research. Room 0.073, Maastricht University, Duboisdomein 30, 6229 GT Maastricht, The Netherlands

## Abstract

**Background:**

While the role of community mobilization in improving maternal health outcomes of HIV positive women in sub-Saharan Africa is continuously emphasized, little is known about how legitimate these claims are. The aim of this study is to systematically review the empirical evidence on this issue.

**Methods:**

A systematic search was conducted in PuBMed, Scopus, Web of Science, MEDLINE, COCHRANE, Allied Health Literature, and Cumulative Index to Nursing.

**Results:**

Our search identified 14 publications on the role of community mobilization in maternal care provision in sub-Saharan Africa, including both HIV negative women and women with HIV, that have used experimental research designs. Regarding HIV negative women, literature has demonstrated that community mobilization is a useful strategy for promoting both positive maternal process results and maternal health outcomes. Most of the literature on women with HIV has focused only on demonstrating the causal link between community mobilization and process results. There has been very little focus on demonstrating the causal link between community mobilization and maternal outcomes for women living with HIV. Overall, the results show that while there is some empirical evidence on a causal link between community mobilization and maternal health outcomes for HIV negative women, this kind of evidence is still missing for HIV positive women. Moreover, as shown by the studies, community mobilization as a maternal health strategy is still in its infancy.

**Conclusion:**

Given the gaps identified in our review, we recommend further research with the aim of providing sound evidence on the role of community mobilization in improving maternal health outcomes of women with HIV in sub-Saharan Africa.

## Background

Despite improvements in funding and policy recommendations by international organizations such as World Health Organization (WHO), United Nations (UN), maternal health remains a challenge in low income countries [1–3]. Studies show that on average, in sub-Saharan African countries, 720 women die each day during pregnancy and childbirth from causes which are largely preventable [4]. The worst maternal health outcomes are found in sub-Saharan African countries [1, 5]. A combination of vulnerabilities and health system weaknesses including poor health infrastructure, poverty, inequality, and HIV are seen as the causes of high maternal mortality [5]. Further, women living with HIV are four times more likely to die during pregnancy and childbirth than HIV negative women [6]. HIV positive women suffer intersecting vulnerabilities. The combined burden of pregnancy-related complications and HIV status is intensified by stigma and discrimination, which prevent some women from seeking help [7].

Intersecting vulnerabilities are further compounded by the dominance of the biomedical approach in handling maternal health challenges [8]. This approach undermines the potential benefits of local knowledge and strategies [9]. Some scholars contend that lack of community involvement led to failed maternal health interventions in sub-Saharan Africa, including maternal health of women living with HIV [10]. There is a traditional tendency inherent in dominant maternal health approaches to undermine local competencies. More specifically, as an antidote to deplorable maternal health outcomes in poor sub-Saharan African settings, mainstream researchers have continued to recommend conventional solutions such as infrastructure improvement, construction of more hospitals and roads, and promotion of women’s rights [11, 12]. While in theory there is no contention on the usefulness of such conventional solutions, evidence has indicated that in practice, little can be done to operationalize these ideal solutions in a sustainable way in poor settings [10]. This is partly because most of these recommendations are costly and thus the majority of the resource-poor governments have been reluctant to implement them. As a result, these solutions remain largely idealistic, especially when applied while ignoring local experience.

Despite the dominant approach to maternal care, several studies have drawn attention to the role of communities and have reported on how involvement of communities, use of indigenous resources and reliance on peer support help to improve health outcomes [2, 13–16]. This observation has ignited the hypothesis that capitalizing on community competencies can complement conventional maternal health efforts especially in sub-Saharan Africa where health systems are inefficient and sometimes inaccessible to many women living with HIV in the peripherals of the societies [17–19].

This is why community mobilization is increasingly being seen as a useful complement to conventional maternal health initiatives [13, 20]. Some examples of initiatives that rely on community mobilization include: a) the “MaiKhanda programme” in Malawi, which is a comprehensive locally-run maternal care project where Traditional Birth Attendants (TBAs) provide maternal services to communities [21]; b) the “Zambulance project”, which connects rural Zambian villages to health centers through a locally manufactured bicycle with an attached trailer used to carry patients and pregnant women to remote health centers [22]; c) the “MaiMwana” project in Malawi, which is an intervention aimed at providing peer support to mothers in rural Malawi [21].

Specific to women living with HIV, Rifkin (2012) also argues that in the absence of an exchange of perspectives and views between professionals and communities, maternal health initiatives for women living with HIV in poor sub-Saharan African communities will continue to meet inaction, mistrust, resistance and failure. It is, however, not known to what extent community mobilization can be a useful strategy for women living with HIV [5, 6]. It is not clear which components of community mobilization actually change conditions among women living with HIV nor how this change is achieved [13, 23].

The aim of this study is to provide an overview of the empirical evidence on the role of community mobilization in maternal care provision to women living with HIV in sub-Saharan Africa. We only include studies with an experimental design in order to identify causal effects. Strong evidence for causal inference is a useful basis for establishing definitive conclusions [24]. The evidence on the role of community mobilization in maternal care provision in general is also reviewed.

Our review is imperative for both policy and research. Researchers have hypothesized that through the generation of locally acceptable, feasible, useful and relevant knowledge and experience, including partnerships with health professionals, community mobilization might be the missing link in the improvement of maternal health [14]. Therefore, our review provides a basis for comparing the role of community mobilization in maternal care provision to HIV negative women and women living with HIV. We also highlight specific areas that need further research. The review can help to guide policy and practice in designing maternal health interventions which are relevant, feasible and accessible to local communities in sub-Saharan Africa [5]. The paper further adds to the gap in literature about how to best operationalize the concept of community mobilization in the context of sub-Saharan Africa.

### Definition of Community Mobilization

Although community mobilization is seen as critical in maternal health promotion, there is little agreement on what defines community mobilization. This is partly because of the diversity in theoretical roots in the development of the concept of community mobilization. This includes Coleman’s (1988), Bourdieu (1986) and Putnam’s (2000) theory of social capital, Michael Foucault’s (1977) theory of governmentality, Jovchelovitch’s (2008) theory of Cognitive Polyphasia and Paulo Freire’s theory of “conscientisation”. In general, the theoretical roots of community mobilization suggest that this concept is premised on the principles of equity, empowerment, inclusion in decision-making, social justice, attention to community, respect for diversity and collaboration [20, 23, 25]. These principles work as core-values in different definitions of community mobilization. Different scholars attach different levels of importance to different principles in their definitions. An overview of definitions of community mobilization in maternal health research is provided in Table 1.

**Table 1:** Summary of community mobilization definitions

Citation	Definition	Central theme
[42]	“A process of creating and harnessing the agency of the marginalized groups most vulnerable to HIV/AIDS, enabling them to build a collective, community response, through their full participation in the design, implementation and leadership of health programmes and by forging supportive partnerships with significant groups both inside and outside of the community”	Campbell et al. (2010) - Community participation - Collaboration between community and professionals - Identity - Indigenous actors
[45]	“Community mobilization is defined as a component of externally- triggered HIV interventions, rather than including indigenous community mobilization initiated by grassroots actors with broader interests than HIV.”	Cornish et al (2014). - Collaboration between community and professionals
[46]	“A participatory approach, which involves building on local competences and strategies by ensuring that community members take part in decision- making and bring local knowledge, experiences and problems to the fore.”	Tripathy et al (2012) - Use of local knowledge - Community participation - Use of indigenous resources
[25]	A capacity building process through which community members, groups and organizations plan, implement and evaluate on a participatory and sustained basis to improve their health or other conditions either on their own initiative or stimulated by others	Howard- Grabman et al (2007) - Local initiatives - Capacity building - Community participatory
[47]	“Community mobilization constitutes active involvement of the community in information sharing , consultation, collaboration and empowerment strategies aimed at bringing change in communities”	Rifkin (2001) - Use of indigenous strategies - Collaboration - Community involvement - Community mobilization as a process/continuum
[48]	“Community mobilization is seen as health promotion intervention which helps communities to identify and undertake appropriate actions in relation to shared problems. Further, there are two types of community mobilization strategies: community organization and building, and community advocacy. The latter assumes involving citizens in institutions or decision which have an impact on their lives”	McKenzie (2013) - Health promotion intervention - Cooperation for collective action - Involvement of community - Community advocacy - Community support
[49]	“Community mobilization is seen as a process where people come together to take action on an issue by relaying on enhanced social connectedness and efficacy or the ability to have influence and control over their situation”	Watson- Thompson et al (2008) - Peer support - Community cooperation
[50]	“Community mobilization is defined as individuals taking action organized around specific community issues. It involves community empowerment, community participation, capacity- building, community coalitions, community organization and development”	Kim- Ju et al (2008) - Community involvement - empowerment, - Capacity- building - Community coalitions - Use of community resources
[51]	“Low- cost, participatory, community- based approaches involving women's groups aimed at effectively improving home delivery practices and birth outcomes in a range of settings”	Nahar et al (2012) - Participatory low cost - Community based strategies - Use of community resources
[21]	“A health promotion strategy best seen as a continuum of process which include, Community informed of decisions made, Community consultation about decisions tokenistically to gain buy in, Community’s views taken into account, Joint decision- making , and Community driven decision- making”	Rosato et al (2006) - Tokenistic - Cooperation - Community consultation - Joint effort - Support
[52]	“Community mobilization is the process of engaging communities to identify community priorities, resources, needs and solutions in such a way as to promote representative participation, good governance, accountability and peaceful change”	Mercy- corps (2009) - Community engagement - Use of Community resources - Community priorities - Community strategies
[19]	“Actions that engage and galvanize community members to take action towards achieving a common goal”	Lippman et al (2013) - Engaging community members - Galvanizing community members

From Table 1, it is clear that any operationalization of the definition of community mobilization should reflect the common principles identified above such as community participation, community empowerment, use of community competence, use of indigenous resources, collaboration, community cooperation, use of peer-support. That notwithstanding, most of these principles are related or just synonyms of each other; thus, in general, they can be grouped into three main ones, namely: (a) *reliance on peer support,* e.g. financial, psychological and social support for pregnant women or new mothers by community members.; (b) *use of indigenous resources in maternal care provision*, e.g. service provision by trained traditional birth attendants (TBA); and/or (c) *collaboration between community and professionals in designing maternal care initiatives,* which is seen through collaborative partnerships between health professionals and communities. Taking this into consideration, for the purpose of our review, we define community mobilization as any initiative that is anchored on at least one of these three principles highlighted above. The central theme among the three principles being community ownership in the form of participation in planning and implementation of the initiative regardless of whether the initiative was externally or locally-initiated.

This operationalization of the concept allows us to capture the community constitutes and health outcomes under investigation. Further, due to the lack of a universally agreed upon definition of the concept of community mobilization, we ensured that we captured as much as possible articles with deeper meaning of community mobilization in which community ownership of an intervention was evident from any of the three principles of community mobilization (reliance on peer support, use of indigenous resources in maternal care provision, collaboration between community and professionals in designing maternal care initiatives).

## Methods

To identify studies to include in our review, a search was conducted in PuBMed, Scopus, Web of Science, MEDLINE, COCHRANE, Allied Health Literature, and Cumulative Index to Nursing with the help of a librarian of Maastricht University.. We conducted a search using the following key-words: community mobilization and maternal health. In addition to this, synonyms of the above phrases were added based on our exploration of the definitions of community mobilization highlighted in Table 1. A complete chain of words used in the search process can be found in Table 2. The search was conducted in April 2017.

**Table 2:** Search strategy

Community mobilization OR community networks OR community groups OR Community coalitions OR community-based OR participatory OR Traditional birth attendants OR community mobilization OR community OR Community engagement OR indigenous strategies OR Local initiatives OR collective action
**AND** [postnatal care OR Prenatal OR ANC OR emergency obstetrics care OR maternal mortality OR complications during pregnancy OR duration of postpartum stay at hospital OR duration of postpartum stay at home OR community care OR Transmission of HIV from Mother to Child]

The procedure included search of articles using different word combinations with the help of a librarian (see Table 2). This was followed by screening of abstracts which lead to exclusion of some papers. Further, thorough reading of retained papers was conducted in order to arrive at the final papers to be included in the study.

To be included in our study, the publications must have:

- reported studies conducted in sub-Saharan Africa
- reported results in English
- reported on community-based initiatives that engaged one or more community groups in concrete participatory activities in designing maternal care initiatives, or used indigenous resources in maternal care provision, or relied on peer support for pregnant women or new mothers.
- have been peer reviewed
- must have made use of an experimental research design, i.e. a randomized trial or a quasi-experimental design..
- must have also been conducted between 1990 and 2015 reflecting the period when HIV became a serious epidemic in sub-Saharan Africa.
- must have evaluated the initiative in terms of process indicators and quantifiable biomedical maternal health outcome indicators as defined by WHO. These include:
  a) *Process indicators for maternal health,* which capture changes in behavior of pregnant women and new mothers. These changes in behavior are usually a direct result of activities of a given maternal health promotion activity and they may include indicators such as: adoption of maternal health-enhancing behavior, increased access to health facility, adherence to antiretroviral therapy if living with HIV, attendance to antenatal and postnatal care, etc. Although useful, these indicators in themselves do not provide information on the results and impact of the activity [2, 26].
  b) *Quantifiable biomedical maternal health outcome indicators*, which capture the eventual health status of the target population and can be divided into [1, 16, 27]: *primary outcomes* (incl. complications during pregnancy and during childbirth, duration of postpartum stay at hospital/community care at home and maternal depression, such as antenatal and postpartum depression); *secondary outcomes* (incl. postnatal care and emergency obstetrical care, maternal mortality)*; other outcomes* (transmission of HIV from mother to child).

**Fig. 1:**
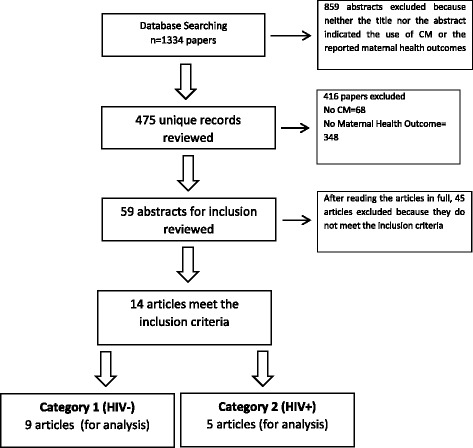
PRISMA Flow Chat for inclusion and Exclusion of articles

The selected publications were then divided into category one studies, which had looked at HIV negative women living in sub-Saharan Africa, and category two studies for women living with HIV in sub-Saharan Africa. The text of the publications was screened based on the same inclusion criteria presented above, and the final list of publications within each category was defined. In Fig. 1, we provide a diagram showing the summary of the search procedure.

We analyzed the text of the publications using content analysis. Content analysis is an approach that helps to synthesize data around key themes. This helps to systematically and objectively describe the study phenomenon [28]. A coding frame was established illuminating the following themes: community mobilization conceptualization, study designs, generalizability of results, process and outcome maternal health indicators. We also followed PRISMA guidelines to ensure quality of our review, see Fig. 1 [29].

## Results

In total, 1334 publications were initially identified, after which 859 publications were excluded because neither the title nor the abstract indicated the use of community mobilization or the reported maternal health outcomes. This left us with 475 publications. After reviewing their abstracts in detail, we excluded another 416 articles leaving us with 59 articles. The full text of the 59 articles were read in detail which led to further exclusion of 46 papers for not meeting the inclusion criteria, thereby leaving us with 14 publications for analysis (Fig. 1). These papers were divided into two categories: those that involved HIV negative women (category one) and those that involved women living with HIV (category two). For category one HIV negative we had nine articles, and for category two women living with HIV, we had five articles. A detailed description of the findings of the publications and their results can be found in Tables 3, 4, 5 and 6.

**Table 3:** Summary of General Characteristics of Result

Characteristic of the publication	Number of publications (%)	Category 1 (HIV-)	Category 2 (HIV+)
Operationalization of Community Mobilization			
-The role of group/peer support for pregnant women or new mothers	5	[8, 17]	[30–32]
-The role of Traditional Birth Attendants (TBA) in maternal care provision	5	[15, 18, 27, 33, 34]	[35]
-The role of partnership between community and professionals in designing initiatives	5	[36–39]	[40]

**Table 4:** Summary of results for HIV Negative (Category 1) and HIV positive women (category 2)

HIV negative (category 1)			
Process outcomes:	Value reported	Number of publications	Publication reference number
Health-enhancing behavioral	Positive change (e.g. increased access to health facility, enhanced the pregnant women’s knowledge on how to handle maternal complications)	6	[15, 18, 27, 33, 36, 37]
Outcome indicators			
Depression rate	Reduced depression	3	[8, 17, 38]
Maternal mortality	Reduced maternal mortality	2	[17] [34]
Hemorrhage/sepsis	Reduced hemorrhage	1	[34]
HIV Positive (category 2)			
Process outcomes:	Value reported	Number of publications (%)	Publication reference number
Health-enhancing behavioral	Positive change: increased maternal health knowledge, Increased utilization and access to health-enhancing resources e.g. antenatal & primary healthcare, PMTCT, information on safe delivery services)	5	[30, 31, 32, 35, 40]
Outcome Indicators			
Depression rate	Reduced depression	1	[30]
Maternal mortality	Reduced maternal mortality	0	
Hemorrhage/sepsis	Reduced hemorrhage	0

**Table 5:** Category 1: Detailed results for HIV negative women

Citation	Country	Study Design	Community Mobilization Component	Process results	Outcome
1. [36]	Tanzania	Longitudinal	Community Capacity Building & Empowerment support for village health workers; (2) developing community-based plans for transportation to health facilities; & (3) increasing participation by community members in planning;	Significant improvement in access t health facilities due to increased transport options Increased attention to Obstetric complications by 275 Deliveries attended by a relative/community member or no one decreased 4% & 6%, respectively but by a TBA increased 200%	
2. [8]	Malawi	randomized controlled trial	Established participatory women’s groups to mobilize communities around maternal and newborn health		A combined community and facility approach using participatory women's groups and quality improvement at health centers reduced newborn mortality in rural Malawi
3. [17]	Malawi	Randomized trial	Established Community women’s groups to ensure provision of socioeconomic support	Better health outcomes for infants reduction in disease for both mother and child Better health-seeking behavior Increased Uptake of HIV testing	Reduced MMR, NMR and IMR in treated group, Conclusion: Community mobilisation through women's groups and volunteer peer counsellor health education are methods to improve maternal and child health outcomes in poor rural populations in Africa.
4. [37]	Kenya	Randomized control trials	Involvement of Community Health Workers in provision of maternal health services	Increased visits to health center, increased deliveries by skilled personnel, “Conclusion increase in essential maternal and neonatal care practices	
5. [38]	Ethiopia	randomized controlled trial	Mother to Mother support in recognizing maternal health risks		Reduced MMR, NMR and IMR in treated group
6. [34]	Angola	longitudinal	Involvement of traditional birth attendants (TBAs) prenatal, delivery, and postnatal care		Better MMR, NMR and IMR outcomes
7. [33]	Sahel	Longitudinal	Involving TBAs to promote safe motherhood	- High levels of retained knowledge of risk factors, hygiene and malaria prophylaxis in 2-year followup survey. - Low levels of knowledge of postpartum haemorrhage management, low number of births attended for most”	
8. [27]	Nigeria	Longitudinal	“Involved traditional birth attendants (TBAs) in a rural community maternal health care provision	- Increased referrals to health centers increased use of family planning	Reduced haemorrhage, oedema, extended labour cases
14. [18]	Sudan	Longitudinal	Involvement of Village TBAs to detect high-risk pregnancy and newborns complications	Increased reporting of complications Increased detection of complications	25% reduction in cases of stillbirth and neonatal death

**Table 6:** Category 2: Detailed results for women living with HIV

Citation	Country of origin	Study Design	Community Mobilization Component	Process results	Outcome
1. [30]	South Africa	Randomized Controlled Trial	Involvement of fellow HIV positive pregnant women to mobilize and provide support to fellow HIV positive mothers.	- Women in Treatment Group (TG) exhibited Increased knowledge on how to improve the health of their children - Increased access to information and resources to improve their health during maternity	- Mothers in Treatment Group (TG) exhibited reduced maternal depression when compared to their counter-parts
2. [31]	Tanzania	Randomized controlled trial	Creation of peer support group that provided a safe space for women with high levels of psychological distress to discuss and share strategies for addressing common concerns related to PMTCT among HIV positive pregnant women.	- Increased disclosure among HIV positive pregnant women in the treatment group as compared to those in the control group. - Women in Treatment Group experienced a significantly higher rate of overall personal satisfaction with response to disclosure,	- a marginally significant reduction in the level of depressive symptoms for HIV-positive pregnant women participating in a group counseling intervention (Treatment Group) 60% of women in the intervention group were depressed post-intervention, versus 73% in the control group [Relative Risk (RR) 0.82, 95% confidence interval (CI): 0.671.01, p0.066]. H
3. [32]	South Africa	cluster randomized controlled	The intervention consisted of four antenatal and four postnatal small group sessions led by Peer Mentors, in addition to Standard Care. HIV positive pregnant Women were recruited during pregnancy and 70 % were reassessed at 1.5 months post-birth.	Compared to Standard Care WLH, EI (treatment Group) were more likely to ask partners to test for HIV (OR = 1.84; two-sided *p* = 0.014),	Compared to Standard Care WLH, EI (treatment Group) were more likely to have infants with height-for-age z-score ≥ –2 (OR = 3.30; *p* = 0.006) and were less likely to report depressed mood (OR = 2.55; *p* = 0.003). - Healthcare utilization was similar across conditions. - Significant improvement in PMTCT
8. [40]	Zambia	(Longitudinal )	Healthcare workers (HCWs) and lay providers conduct rapid HIV testing. support for pregnant women,”	Uptake of services significantly improved HIV testing among pregnant women improved - There was also significant improvement in the percentage of HIV positive pregnant women referred for clinical care	

### Overall characteristics of publications

Table 3 presents an overview of the general characteristics of the publications reviewed. The studies were published in peer reviewed journals between 1990 and 2015 and were all based on experimental research designs.

Regarding the definition of community mobilization, overall, the studies operationalized community mobilization in accordance with the three main principles namely; *collaboration between community and professionals in designing maternal care initiatives, use of indigenous resources in maternal care provision (e.g. TBA)*, *reliance on peer support for pregnant women and new mothers*. In total, five publications operationalized community mobilization as group/peer support (social, psychological, and financial), another five operationalized community mobilization as the use of indigenous resources in maternal care provision (e.g. TBAs), and another five conceptualized community mobilization as partnership between community and health professionals in the design and implementation of maternal care initiatives.

### The role of Community Mobilization among HIV negative women (Category 1) and HIV positive women (Category 2)

Table 4 presents a summary of the indicators used to study the process and outcome of community mobilization among both HIV negative women and women living with HIV.

With regard to HIV negative women, some of the publications reported process indicators which involved adoption of health-enhancing behavior among mothers. The others reported outcome indicators, which included reduction in maternal depression, reduced rates of hemorrhage, reduced neonatal mortality and reduced maternal mortality. On the other hand, the majority of publications on women living with HIV reported process indicators, which included among other things, adoption of maternal health-enhancing behavior among HIV positive mothers. In addition, a few reported outcome indicators such as reduced depression among HIV pregnant women.

Peer support was explicated in the form of regular meetings in women’s groups where pregnant women provided each other with emotional, physical, social and psychological support to deal with high levels of psychological distress and other practical needs. They also shared strategies for addressing common concerns related to antenatal and primary healthcare, including Prevention of Mother To Child Transmission (PMTCT) among women with HIV. Although there was more emphasis on support for PMTCT for women with HIV, in general, the type of peer support ( financial, psychological, and material) given was to a large extent similar for both HIV negative and women positive women [8, 17, 30–32].

For HIV negative women, peer support improved emotional strength and enhanced pregnant women’s knowledge on how to handle maternal complications such as antepartum and postpartum hemorrhage, obstructed labor, and sepsis [8]. Further, peer support arising from participation in women’s groups in the community significantly reduced the risk of mortality among newly born children [8, 17]. Overall, the majority of publications in this systematic review concluded that peer support in HIV negative women led to improvements in maternal and child health outcomes due to improved access to emotional, psychological, social and physical support, which enabled pregnant women to access information, services and resources that are necessary for improving maternal health outcomes .

Three publications regarding women with HIV reported that peer support among other things, helped to increase access to health-enhancing resources (such as antenatal and primary healthcare, PMTCT, information on safe delivery services) and knowledge, it also increased utilization of health services by providing pregnant women with transport to health centers and safe delivery packages. Women with HIV who had peer support also experienced reduced maternal depression [30–32].

- Use of traditional birth attendants (TBA) in maternal care provision

The studies that reported on TBAs demonstrated how TBAs helped to increase women’s knowledge on risk factors during pregnancy, knowledge on hygiene, use of malaria prophylaxis among HIV negative pregnant women who were outside the catchment area of standard health care [18, 27, 33]. One publication highlighted that the use of TBAs helped to reduce maternal depression, hemorrhage, and maternal mortality among HIV negative women [34]. These studies concluded that in general, TBAs contribute positively to maternal health outcomes of HIV negative women in the peripherals of sub-Saharan Africa.

Among women living with HIV, the only publication that was found concluded that using TBAs enhanced the coverage of maternal health care for women living with HIV especially in areas where many pregnant women chose to have home deliveries [35]. TBAs were considered useful because they encouraged women with HIV to take medication (e.g. zidovudine), which helped to prevent mother-to-child transmission of HIV during pregnancy. The evidence examined in this systematic review however does not report exactly on whether and how the TBAs managed to deal with complications such as hemorrhage, sepsis and PMTCT. The only study on women living with HIV was also silent on what mechanisms were available to TBAs for referral purposes in cases of emergency.

- Collaboration between community and professionals in designing and/or implementing initiatives

The role of collaboration in the design and implementation of maternal care initiatives for HIV negative women was studied in four publications [36–39]. Collaboration helped to design initiatives that improved access to professional treatment. Such initiatives included transportation of pregnant women by community members to health centers in case of obstetric emergencies. This sort of collaboration increased the attention to other complications such as sepsis and hemorrhage. This was because the collaboration allowed for easy exchange of knowledge and information regarding these complications between professionals and the community [36–39]. Further, it was deduced from the publications that through the collaboration between the community and professionals, there was an increase in antenatal services and institutional delivery [36, 37]. Other studies showed that through the collaboration between health workers and local community members, there was a significant reduction in child mortality of up to 57% [38]. Another study from Tanzania concluded that through collaboration with community care-givers, health-inhibiting practices during home deliveries were mitigated (e.g. use of unclean thread to tie the umbilical cord, putting “substances” on the cord ) [39]. Overall, it is evident from the publications that collaboration between professionals and communities in the design of maternal care initiatives improved access to health information, treatment and services, as well as increased attention to maternal health complications, thereby making this a viable strategy in an attempt to improve maternal health outcomes of HIV negative women.

On the other hand, only one publication evaluated the role of community involvement in promoting maternal health of women living with HIV [40]. This study also only reported positive process results in the form of improved uptake of institutional health services and acceptance of maternal health interventions; both of which are assumed to be important in improving maternal health of women living with HIV. It is therefore clear that the focus of research on community involvement and maternal health outcomes of women with HIV was limited to explication of process results only, without much on outcomes. There is still a gap in linking collaboration in maternal care initiatives to outcome results such as reduced maternal mortality, reductions in complications such as sepsis and hemorrhage in women with HIV.

## Discussion

Overall, this systematic review of experimental design studies has produced results which differ somewhat between women living with HIV and HIV negative women. We observe that to date, there is sound causal evidence in support of community mobilization as a useful maternal health care strategy for HIV negative women. On the other hand, strong evidence for women with HIV is still missing. The fact that strong evidence linking community mobilization to maternal health outcomes for women with HIV is still missing confirms Kendall and Langer’s (2015) assertion that the intersection of HIV and pregnancy has generally been ignored in social science research [4]. This is despite the fact that women with HIV are more susceptible to morbidity and mortality during pregnancy [5]. Below we demonstrate in detail the differences in empirical evidence between HIV negative women and women living with HIV using the three principles of community mobilization.

Firstly, evidence indicates that for HIV negative women, *peer support* from fellow pregnant women improves maternal health outcomes. Our findings are consistent with a growing body of literature which shows that peer support is necessary in reducing rates of maternal depression, antepartum and postpartum hemorrhage, obstructed labor and sepsis in HIV negative women [41]. This is normally achieved through provision of economic and psychological support to peers, including sharing of different strategies in order to deal with negative maternal outcomes. It is clear from existing literature that peer support is useful for HIV negative women, however, the same cannot be said of women with HIV. Given the relatively high risk associated with HIV/AIDS, and this coupled with the lack of studies focusing on women with HIV, it remains unclear whether peer support can also be useful in reducing maternal complications in women with HIV, and if so, in which ways. This however does not imply that peer support is completely useless for women with HIV. The indication from our results is that peer support is still useful in promoting process results which include enhanced coping, increased access to emotional and economic support, adherence to treatment, utilization of health services [30–32]. While most scholars attach less importance to processes within different maternal health interventions, it should be noted however that these process outcomes are not completely unrewarding. They embody social conditions that are necessary for maternal health improvement [42]. In particular, the processes observed in this review are important for women living with HIV who are usually victims of isolation, stigma and discrimination. Peer support thus gives rise to social spheres which provide opportunities for critical dialogue about maternal health issues among women with HIV [14, 43]. Peer support also necessitates the re-assessment of harmful cultural and social norms affecting women with HIV [41]. While it is true that in general peer support leads to conditions necessary for improving maternal health in women with HIV, this fact alone does not offer any basis for drawing a causal link between peer support and maternal health of women living HIV. Existing evidence does not provide a knock-on effect of peer support on maternal health outcomes such hemorrhage, sepsis, and maternal mortality for women with HIV. Evidence linking peer support to maternal health outcomes of women with HIV is still missing in literature. The evidence provided in support of peer support vis-à-vis women with HIV seems necessary but not sufficient to unequivocally establish peer support as a useful strategy for improvement of maternal outcomes among women with HIV. More research needs to be undertaken to examine this relationship in order to shade light on whether peer support can actually lead to improved maternal health outcomes among women with HIV, and if so, how exactly this change is achieved.

The other important principle of community mobilization is the involvement of TBAs in maternal care provision. Our results in this regard were concomitant with recent studies indicating that TBAs are particularly useful for enhanced maternal care for HIV negative women, especially in areas lacking efficient health systems [18, 27, 33]. TBAs were particularly useful in reducing maternal morbidity and mortality in HIV negative women. However, this evidence only speaks for HIV negative women, whereas no focus has been placed on women living with HIV. The impact of TBAs on maternal health outcomes in women with HIV (who require antiretroviral therapy and cesarean birth in order to promote PMTCT) remains understudied [35, 44]. There is a need for more research to establish the link between use of TBAs and positive maternal health outcomes in women with HIV. Although a stronger case for TBAs is still lacking, it does not automatically mean that TBAs are irrelevant to women with HIV in sub-Saharan Africa. To the contrary, this result only means that to date, there is no study that establishes a significant causal link between TBAs and maternal health outcome of women with HIV. We also note that in sub-Saharan Africa, it is counterproductive to encourage only institutional delivery. Institutional delivery in poorly equipped, hard-to-reach, remote, and overcrowded health facilities with poor adherence to quality is far from optimal. Given this, TBAs are sometimes the only feasible, cheap, practical and readily available source of maternal care in the peripherals of Africa and as such, it seems more advantageous in the context of sub-Saharan Africa to make use of TBAs and professional midwives complementary to each other [35, 37]. Therefore, a more nuanced understanding of maternal health interventions for women with HIV in sub-Saharan Africa is needed. However, before achieving this, it is vital that we increase our understanding on how exactly TBAs can improve maternal health of women with HIV and what kind of training should be provided to them.

Lastly, collaboration between professionals and the community in the design and implementation of maternal care initiatives, which is another principle of community mobilization, also produced varied results for HIV negative women and for women with HIV. The causal link between collaboration and maternal health outcomes for HIV negative women is established [36–39]. To date, collaboration with regard to women with HIV is only credited for the adoption of health-enhancing behavior. Although not a maternal health outcome in itself, behavioral change is an essential step towards improving maternal health. At the same time, it should be noted that behavioral change does not epitomize improved maternal outcome, it is merely a process through which improvement in maternal health can be achieved. This means that currently, it is less lucid to claim a collaboration-precipitated maternal health improvement in women with HIV on the basis of behavioral change. Therefore, we posit that more evidence is needed to understand how useful collaboration could be for women with HIV. Thus, before advocating for collaboration as a panacea to maternal health challenges among women with HIV in sub-Saharan Africa, there is a need for more research which establishes a causal link beyond behavioral change; one which can clearly show the effect of collaboration on maternal health outcomes in women with HIV.

Our review has several limitations that need to be acknowledged. Key among them is the fact that the search only included publications in English. This may have led to the omission of studies published in other languages within sub-Saharan Africa. The search also just focused on studies published in peer-reviewed journals thereby leaving out any grey literature. Therefore, it is possible that some literature focusing on the role of community mobilization on maternal health outcomes of HIV positive women in sub-Saharan Africa may have been omitted. We also did not find studies reporting undesirable process or outcome results. It should be acknowledged in this regard that positive results are more easily published while negative results tend to remain underreported.

## Conclusion

As shown in our systematic review, currently, the evidence on the role of community mobilization on maternal outcomes of HIV negative women in sub-Saharan Africa is more conclusive than for women with HIV. This is embodied in all principles of community mobilization that we analyzed. So far, not much empirical work has been done to provide compelling evidence regarding the impact of community mobilization on maternal outcomes (i.e. sepsis, hemorrhage, and maternal mortality) for women living with HIV. That notwithstanding, there is some evidence suggesting that all three principles of community mobilization provide necessary process results for improving maternal health of women with HIV. It is indeed true that the process results create necessary conditions for improving maternal health. However, evidence on process indicators alone without subsequent causal evidence on the outcomes is not sufficient, and as such, cannot be used as a basis for advocating community mobilization as a useful maternal health care strategy for women with HIV in sub-Saharan Africa. Thus, more research needs to be undertaken to examine whether community mobilization actually leads to better maternal health outcomes in women with HIV and how exactly this change is achieved. Consequently, when it comes to relying on community mobilization for policy recommendation, we observe that there is not enough evidence to recommend community mobilization as a strategy for maternal health care provision for women in sub-Saharan Africa.
